# Virtual Clinic Telehealth Abortion Services in the United States One Year After Dobbs: Landscape Review

**DOI:** 10.2196/50749

**Published:** 2024-08-05

**Authors:** Leah R Koenig, Jennifer Ko, Ushma D Upadhyay

**Affiliations:** 1 Advancing New Standards in Reproductive Health Department of Obstetrics, Gynecology & Reproductive Sciences University of California, San Francisco Oakland, CA United States; 2 Center for Gender and Health Justice Global Health Institute University of California Oakland, CA United States; 3 Department of Epidemiology and Biostatistics Univeristy of California, San Francisco San Francisco, CA United States

**Keywords:** medication abortion, telehealth, virtual clinics, abortion, access, policy, health equity

## Abstract

**Background:**

Telehealth abortion has taken on a vital role in maintaining abortion access since the *Dobbs v. Jackson Women’s Health Organization* Supreme Court decision. However, little remains known about the landscape of new telehealth-only virtual clinic abortion providers that have expanded since telehealth abortion first became widely available in the United States in 2021.

**Objective:**

This study aimed to (1) document the landscape of telehealth-only virtual clinic abortion care in the United States, (2) describe changes in the presence of virtual clinic abortion services between September 2022, following the *Dobbs* decision, and June 2023, and (3) identify structural factors that may perpetuate inequities in access to virtual clinic abortion care.

**Methods:**

We conducted a repeated cross-sectional study by reviewing web search results and abortion directories to identify virtual abortion clinics in September 2022 and June 2023 and described changes in the presence of virtual clinics between these 2 periods. In June 2023, we also described each virtual clinic’s policies, including states served, costs, patient age limits, insurance acceptance, financial assistance available, and gestational limits.

**Results:**

We documented 11 virtual clinics providing telehealth abortion care in 26 states and Washington DC in September 2022. By June 2023, 20 virtual clinics were providing services in 27 states and Washington DC. Most (n=16) offered care to minors, 8 provided care until 10 weeks of pregnancy, and median costs were US $259. In addition, 2 accepted private insurance and 1 accepted Medicaid, within a limited number of states. Most (n=16) had some form of financial assistance available.

**Conclusions:**

Virtual clinic abortion providers have proliferated since the *Dobbs* decision. We documented inequities in the availability of telehealth abortion care from virtual clinics, including age restrictions that exclude minors, gestational limits for care, and limited insurance and Medicaid acceptance. Notably, virtual clinic abortion care was not permitted in 11 states where in-person abortion is available.

## Introduction

Following the 2022 *Dobbs v. Jackson Women’s Health Organization* US Supreme Court decision, demand for abortion surged in states where abortion care remains legally accessible [1]. Telehealth abortion has emerged as an important model of abortion provision in the US in recent years. In 2021, the US Food and Drug Administration (FDA) lifted a restriction that required mifepristone, the first drug in the medication abortion regimen, to be dispensed only inside medical facilities, thus allowing direct-to-patient telehealth abortion care to expand. As of December 2023, telehealth constituted 19% of abortions in the US health care system [1].

Telehealth reduces geographic barriers to abortion and decreases wait times to care [2-4]. However, nearly all Southern and Midwest states ban abortion entirely or permit abortion but prohibit telehealth for abortion, limiting telehealth’s potential to help maintain abortion access. Such restrictions on telehealth include in-person counseling, ultrasound, or other testing requirements and prohibitions of telehealth for abortion [5].

Direct-to-patient telehealth abortion is safe and effective [6-9]. Patients typically complete synchronous (over videoconferencing or a phone call) or asynchronous (using secure messaging) screening with a clinician to assess for medical eligibility. Once deemed eligible, patients are mailed medications, typically from a mail-order pharmacy. They then take the medications, pass the pregnancy, and complete follow-up interactions with their provider, from home or another place they choose [10].

Virtual abortion clinics—telehealth abortion providers without brick-and-mortar facilities in the state where they are providing abortion care—have also proliferated. However, because virtual abortion clinics are so new, little is known about their availability, reach, and policies. We aimed to document the landscape of virtual clinic abortion care in the US, to describe changes in the availability of virtual abortion clinics over time between September 2022, just after the *Dobbs* decision, and June 2023, and to identify structural factors that may perpetuate inequities in access to virtual clinic abortion care.

## Methods

This repeated cross-sectional landscape review involved web searches (search terms listed in Textbox 1) and 3 abortion directory websites (Abortionfinder, Ineedana, and Plan C) that document abortion service availability. We synthesized all available information from the 3 abortion directories and clinic websites.

Virtual clinics were eligible if they provided telehealth abortion care within the US health care system during the search period. We included virtual clinics that provided telehealth abortion services in states where they did not have a brick-and-mortar location.

LRK and JK conducted the first and second searches in September 2022 and June 2023, respectively. They conducted independent searches to identify eligible virtual clinics and document their policies. Discrepancies were then resolved iteratively through a third collaborative search.

Our primary measures of interest were the number of unique virtual clinics operational at the time of each search and the number of states served. These were assessed in both September 2022 and June 2023. Our secondary measures of interest were the service policies of each virtual clinic, including ages served, costs, insurance and Medicaid acceptance, gestational limits, languages offered, whether the service offered synchronous care (involving video or phone interactions) and/or asynchronous care (entirely over secure messaging), and whether the virtual clinic provided medication abortion through advanced provision. These were assessed only in June 2023. We described each outcome using descriptive statistics including frequencies, percentages, medians, and modes. We adhered to the Strengthening the Reporting of Observational Studies in Epidemiology (STROBE) reporting guidelines.

**Search terms.**

“telehealth”

“telemedicine”

“online”

“abortion pills”

“medication abortion”

“mifepristone”

“misoprostol”

## Results

**Figure 1:**
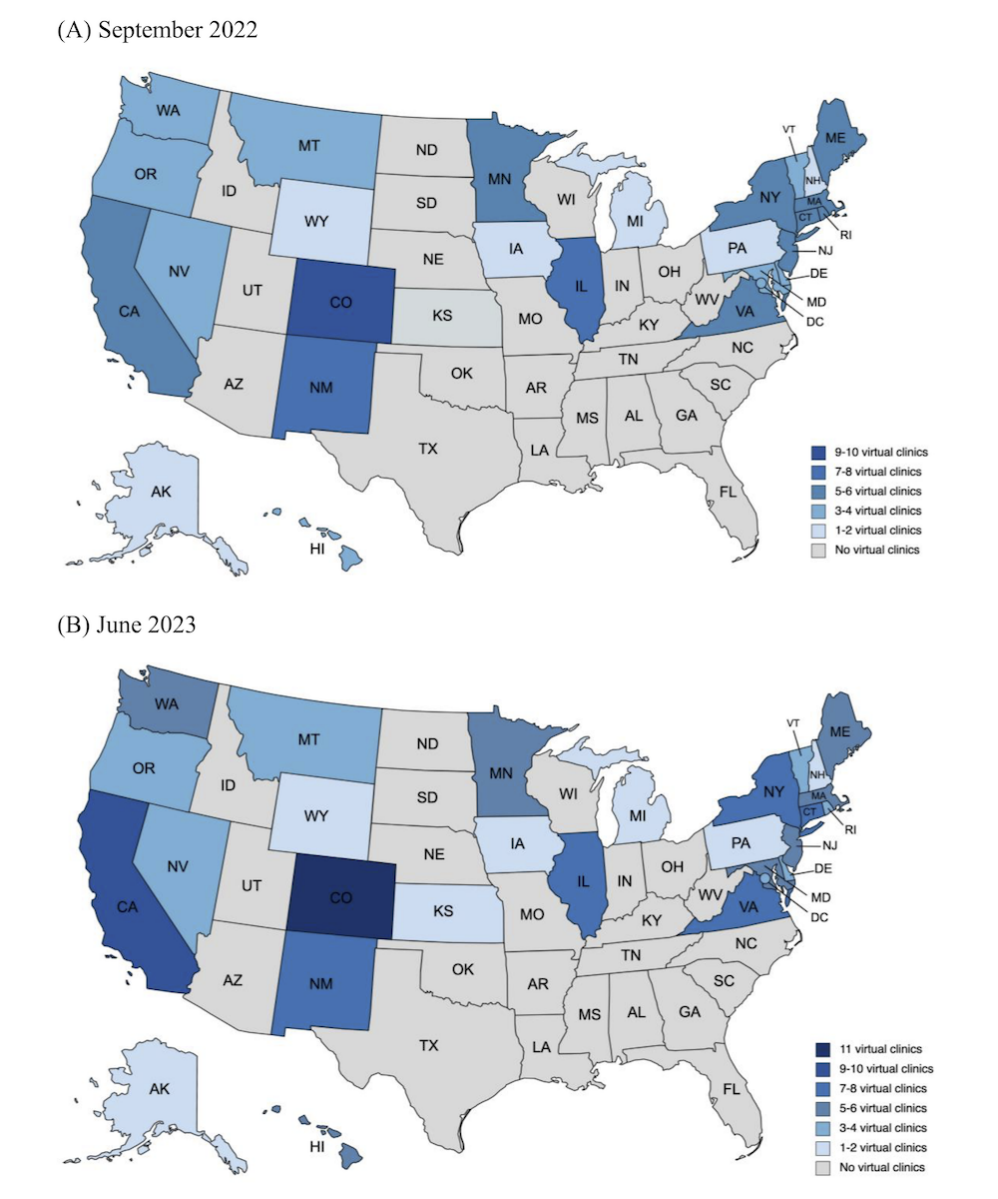
Map of virtual clinic telehealth abortion care availability in the United States, September 2022 (A) and June 2023 (B). The figure was created with mapchart.net.

In September 2022, 11 virtual clinics provided telehealth abortion care in 26 states and Washington DC **(**Table S1 in Multimedia Appendix 1). By June 2023, 20 virtual clinics provided telehealth abortion care in 27 states and Washington DC (Figure 1). In June 2023, 23 states had no virtual clinic providers, among which 14 had banned abortion entirely and 9 allowed abortion but had restrictions on telehealth for abortion.

In June 2023, gestational limits ranged from 10 weeks, 0 days to 13 weeks, 0 days, although the modal gestational limit was 10 weeks, 0 days (n=8; Table 1). Among the 20 virtual clinics, 4 did not serve patients younger than 18 years. Overall, 8 virtual clinics provided only synchronous care, 6 provided asynchronous care, and 6 offered patients a choice between the two. In addition, 6 offered medication abortion by advanced provision.

Costs for virtual clinic telehealth abortion care ranged from US $90 to US $600, and median costs were US $259. Few (n=2) accepted private insurance and 2 accepted Medicaid in certain states. Most virtual clinics (n=15) offered some form of financial assistance to patients. About half offered services in languages other than English (n=11). However, while some of the virtual clinic providers were multilingual, many platforms were only available in English, and some charged additional costs for translations.

**Table 1:** Availability and policies of virtual abortion clinics in the United States, June 2023.

Clinic name	Gestational limit	Telehealth care model	Age limit	Languages offered	Advanced provision	Cost (in US $)	Accepted private insurance	Accepted Medicaid	Financial assistance available	States served
145 Abortion Telemedicine	13 weeks 0 days	Synchronous	None	Over 500 languages with translational service (US $100)	No	145	No	No	No	18 (CA, CO, CT, DE, DC, HI, IL, ME, MA, MT, NJ, NM, NY, OR, RI, VT, VA, and WA)
Aid Access	12 weeks 0 days	Asynchronous	13+	English	Yes	150	No	No	Yes	20 (AK, CA, CO, DC, HI, IL, ME, MD, MA, MI, MN, NV, NJ, NM, NY, OR, RI, VT, VA, and WA)
Abortion on Demand	10 weeks 0 days	Synchronous	18+	English	No	239-389	No	No	Yes	24 (CA, CO, CT, DE, DC^a^, HI, IL, KS, ME, MD, MA, MN, MT, NV, NH, NJ, NM, NY, OR, PA^a^, RI, VT, VA, and WA)
carafem	11 weeks 0 days	Synchronous	None	Spanish translator available by phone	No	249	No	IL only	Yes	15 (CO, CT, DE, DC, IL, IA, ME, MA, MN, NV, NJ, NM, RI, VT, and VA)
Choices Rising	10 weeks 0 days	Asynchronous	15+	English, Spanish	No	297	No	CA only	No	2 (CA and HI)
Choix	10 weeks 5 days	Asynchronous	15+ (certain states)	English, Spanish	Yes	399	No	No	Yes	6 (CA, CO, IL, ME, NM, and VA)
Forward Midwifery	12 weeks 3 days	Synchronous or asynchronous	None	English, Spanish, Translation Services	No	150	No	No	Yes	6 (CA, CO, MD, MA, NM, and OR)
Hey Jane	10 weeks 0 days	Synchronous or asynchronous	18+	English	No	199-409	Yes	No	Yes	9 (CA, CO, CT, IL, NJ, NM, NY, VA, and WA)
Jennifer Boyd	10 weeks 0 days	Synchronous	None	English	No	550	No	No	Yes	2 (CT and NY)
Juniper Midwifery	11 weeks 0 days	Synchronous or asynchronous	None	English, Spanish	Yes	200	No	No	Yes	6 (CA, CO, CT, NJ, NM, and NY)
Just the Pill	10 weeks 0 days	Synchronous	None	English, Spanish	No	350	No	No	Yes	4 (CO, MN, MT, and WY)
Lilith Care	12 weeks 0 days	Asynchronous	18+	English	No	140	No	No	No	3 (HI, MA, and RI)
Luna Flow Health	10 weeks 6 days	Synchronous	13+	English	Yes	195-395	No	No	Yes	1 (CA)
Maitri Wellness	12 weeks 0 days	Synchronous or asynchronous	18+	English, Hindi, Spanish	Yes	150-500	No	No	No	3 (MI, NJ, and NY)
Metro Area	12 weeks 6 days	Synchronous or asynchronous	None	English	Yes	165	Yes	No	Yes	3 (DC, MD, and VA)
Joan Fleischman	10 weeks 0 days	Synchronous	None	English	No	600	No	No	Yes	3 (CT, NJ, and NY)
Pills by post	12 weeks 0 days	Asynchronous	14+	English, Spanish	No	150	No	No	Yes	4 (CO, IL, MN, and NY)
The Satanic Temple	11 weeks, 0 days	Synchronous	17+	English	No	90	No	No	Yes	1 (NM)
Sunny	10 weeks 0 days	Asynchronous	16+	English, Spanish, Translation Services	No	269	No	No	No	2 (CA and NY)
Wisp	10 weeks 0 days	Synchronous or asynchronous	18+	English	No	200	No	No	Yes	8 (CO, CT, IL, ME, MD, NM, NY, and WA)

^a^Telehealth abortion care available with in-person pickup in a neighboring state for certain states.

## Discussion

### Principal Findings

Virtual clinics increased dramatically in the United States between 2022 and 2023, the year after the *Dobbs* decision; they doubled in number and increased service provision to reach 27 states and Washington, DC. Our results show that telehealth abortion is an important and rapidly expanding model of abortion care.

We documented several structural barriers in the landscape of virtual clinic abortion that could be addressed through changes in virtual clinic policies. Most virtual clinics we examined offered medication abortion care up to 10 weeks of pregnancy, although medication abortion is commonly provided off-label in the United States until 11 weeks and is recommended for use by the World Health Organization up to 12 weeks [11,12]. As abortion bans created additional logistical hurdles that delay abortion care, virtual clinics should expand gestational limits to serve patients throughout the first trimester. Future research should assess the safety of telehealth abortion provided at gestations beyond 11 weeks.

Some virtual clinics had minimum age requirements that are not legally mandated in the states they serve. These restrictions limit abortion access for adolescents, who face even greater barriers to abortion and stand to benefit from the privacy and ability to avoid travel that telehealth offers [13]. In addition, few virtual clinics accepted private insurance or Medicaid, highlighting an important accessibility gap. Most virtual clinics we identified offered only asynchronous or synchronous care. Research has demonstrated that both are safe and effective and that each offers unique benefits to patients [4,7,9]. It is also critical that virtual abortion clinics offer multilingual services to reach immigrant and undocumented populations—groups for whom travel for abortion care may be especially difficult. Future qualitative research can highlight changes needed to enable virtual clinics to remove these barriers to care.

We also identified state policy changes that could help reduce inequities in telehealth abortion care. First, states can take action to improve equitable access to telehealth medical care more broadly through actions such as increasing coverage for asynchronous telehealth care and promoting equitable access to reliable internet connection. Second, they can promote access to telehealth abortion by improving Medicaid and insurance reimbursement [14]. Third, in-person counseling, ultrasound, and other requirements are not based on medical evidence, and states should remove them to allow telehealth abortion in all states where abortion is legal.

This analysis had several limitations. First, we did not document virtual clinic policies during the first search in September 2022, and therefore we could not examine changes in these policies over time. Second, we may have missed virtual abortion clinics not documented on the websites we examined or in our searches. Third, in some cases, the policies listed on virtual clinic websites or abortion directories may differ from actual practice. For example, advance provision may be available from providers who did not publicize those services. Fourth, this review does not encompass telehealth services provided by brick-and-mortar clinics. However, these results provide a novel picture of the landscape of telehealth abortion care from virtual clinics in the United States in the year following the *Dobbs* decision. By conducting a landscape review of the information available from web searches and abortion directories, we provide a sense of the information available to prospective abortion patients across the United States.

Virtual clinics are emerging as key providers in the US abortion landscape in the face of dire restrictions on abortion access. In 2022 and 2023, several states that protect abortion passed “shield laws,” which create legal protections for clinicians providing telehealth abortion to patients in states with bans [15]. These laws are further expanding the role of telehealth by allowing patients residing in states where abortion is banned to access telehealth abortion care within the US health care system. Since our review in June 2023, several virtual clinics have begun to offer telehealth abortion care to residents of states with abortion bans under these shield laws [16,17]. However, as long as patients experience legal risks using such services, access will never be equitable. To ensure health equity, all people should have access to abortion care offered by virtual clinics regardless of the state they live in.

### Conclusion

Virtual abortion clinics have grown in prominence in the US since 2022. As of 2023, many virtual clinics retained policies that may limit equitable access to abortion care, such as minimum age requirements and gestational limits not required by law and lack of insurance acceptance. Given the increasingly restricted US abortion landscape, it is critical to address barriers to telehealth abortion care to ensure as many people as possible can access abortion care.

## Appendix

Multimedia Appendix 1 Availability of virtual abortion clinics in the United States, September 2022.

## Abbreviations

FDA: US Food and Drug Administration
STROBE: Strengthening the Reporting of Observational Studies in Epidemiology
