# Supplementary material for: Virtual Clinic Telehealth Abortion Services in the United States One Year After Dobbs: Landscape Review
Source: J Med Internet Res. 2024 Aug 5;26:e50749. doi: 10.2196/50749 (PMC11333862; doi:10.2196/50749)
Supplement: Multimedia Appendix 1 [file jmir_v26i1e50749_app1.docx]

**Table S1**

| **Clinic name** | **States served** |
| --- | --- |
| **145 Abortion Telemedicine** | 18: CA, CO, CT, DE, DC, HI, IL, ME, MA, MT, NJ, NM, NY, OR, RI, VT, VA, WA |
| **Aid Access** | **21: AK,** CA, CO, CT, DC**, HI,** IL, ME, MA**, MD, MI, MN, NY, NJ, NM, NV, OR, RI, VT, VA, WA** |
| **Abortion on Demand** | 23: CA, CO, CT, DE, DC^a^, HI, IL, ME, MD, MA, MN, MT, NV, NH, NJ, NM, NY, OR, PA^a^, RI, VT, VA, WA |
| **carafem** | 15: CO, CT, DE, DC, IL, IA, ME, MA, MN, NV, NJ, NM, RI, VT, VA |
| **Choix** | 6: CA, CO, IL, ME, NM, VA, |
| **Forward Midwifery** | 6: CA, CO, MD, MA, NM, OR |
| **Hey Jane** | 8: CA, CO, IL, NJ, NM, NY, VA, WA |
| **Jennifer Boyd** | 2: CT, NY |
| **Just the Pill** | 4: CO, MN, MT, WY |
| **Lillith Care** | 3: HI, MA, RI |
| **Pills by post** | 4: CO, IL, MN, NY |
| a – Abortion on Demand offers telehealth abortion care via in-person pickup in a neighboring state in Pennsylvania and Washington, D.C. | |
